# Characterization of two multidrug-resistant *Klebsiella pneumoniae* harboring tigecycline-resistant gene *tet*(X4) in China

**DOI:** 10.3389/fmicb.2023.1130708

**Published:** 2023-04-26

**Authors:** Yanxian Yang, Ruowen He, Yiping Wu, Mingyang Qin, Jieyun Chen, Yu Feng, Runping Zhao, Lei Xu, Xilong Guo, Guo-Bao Tian, Min Dai, Bin Yan, Li-Na Qin

**Affiliations:** ^1^Program in Pathobiology, the Fifth Affiliated Hospital, Zhongshan School of Medicine, Sun Yat-sen University, Guangdong, China; ^2^Advanced Medical Technology Center, The First Affiliated Hospital, Zhongshan School of Medicine, Sun Yat-sen University, Guangzhou, China; ^3^Key Laboratory of Tropical Diseases Control, Ministry of Education, Sun Yat-sen University, Guangzhou, China; ^4^Microbiome Medicine Center, Department of Laboratory Medicine, Zhujiang Hospital, Southern Medical University, Guangzhou, China; ^5^School of Basic Medical Sciences, Xinxiang Medical University, Xinxiang, China; ^6^School of Public Health, Shandong University, Jinan, China; ^7^School of Laboratory Medicine, Chengdu Medical College, Chengdu, China; ^8^Department of Neonatal Surgery, Guangzhou Women and Children's Medical Center, Guangzhou, China; ^9^Faculty of Forensic Medicine, Zhongshan School of Medicine, Sun Yat-sen University, Guangzhou, China; ^10^Guangdong Province Translational Forensic Medicine Engineering Technology Research Center, Sun Yat-sen University, Guangzhou, China

**Keywords:** *Klebsiella pneumoniae*, tigecycline resistance, *tet*(X4), horizontal transfer, swine

## Abstract

**Objectives:**

Tigecycline is recognized as one of the last-line antibiotics to treat serious bacterial infection caused by carbapenem-resistant *Klebsiella pneumoniae* (CRKP). The plasmid-borne gene *tet*(X4) mediates high resistance to tigecycline. However, the prevalence and genetic context of *tet*(X4) in *K. pneumoniae* from various sources are not fully understood. Here, we investigated the prevalence of *tet*(X4)-positive *K. pneumoniae* and characterized the genetic context of *tet*(X4)-bearing plasmids in *K. pneumoniae* isolates.

**Methods:**

Polymerase chain reaction (PCR) was used to detect the *tet*(X4) gene. The transferability of the *tet*(X4)-carrying plasmids was tested by conjugation assays. The *Galleria mellonella* infection model was used to test virulence of *tet*(X4)-positive strains. Whole-genome sequencing and genome-wide analysis were performed to identify the antimicrobial resistance and the virulence genes, and to clarify the genetic characteristics of the *tet*(X4)-positive isolates.

**Results:**

Among 921 samples, we identified two *tet*(X4)-positive *K. pneumoniae* strains collected from nasal swabs of two pigs (0.22%, 2/921). The two *tet*(X4)-positive isolates exhibited high minimum inhibitory concentrations to tigecycline (32–256 mg/L) and tetracycline (256 mg/L). The plasmids carrying the *tet*(X4) gene can transfer from the donor strain *K. pneumoniae* to the recipient strain *Escherichia coli* J53. Genetic analysis of the complete sequence of two *tet*(X4)-carrying plasmids pTKPN_3-186k-tetX4 and pTKPN_8-216k-tetX4 disclosed that the *tet*(X4) gene was flanked by delta IS*CR2* and IS*1R*, which may mediate the transmission of the *tet*(X4) gene.

**Conclusion:**

The prevalence of *tet*(X4)-positive *K. pneumoniae* among different sources was low. IS*CR2* and IS*1R* may contribute to the horizontal transfer of *tet*(X4) gene. Effective measures should be taken to prevent the transmission of *tet*(X4)-producing *K. pneumoniae* in humans or animals.

## Introduction

*Klebsiella pneumoniae* is a common cause of antimicrobial-resistant opportunistic infections in hospitalized patients (Wyres et al., 2020). In clinical, *K. pneumoniae* can cause a variety of infection diseases, including bacteremia, urinary tract infections, pneumonia, and liver abscesses (Hansen et al., 1998; Siu et al., 2012). In recent years, carbapenem resistant *K. pneumoniae* (CRKP) infection rates have increased alarmingly (Low et al., 2018). Tigecycline has been regarded as one of the “last resort” antimicrobials to fight against CRKP (Seifert et al., 2018).

Tigecycline is the first generation of glycylcycline, which has been used since 2005. It is one of the last choice of treatment for serious infection, especially those caused by extensively drug-resistant Enterobacteriaceae (Sun et al., 2019). Shortly after the first usage, a multi-drug resistant (MDR) *K. pneumoniae* strain (reduced tigecycline sensitivity, MIC = 4 μg/ml) was isolated in a hospital, significantly compromising the efficacy of tigecycline (Ruzin et al., 2005). To date, there are several known mechanisms associated with tigecycline resistance in *K. pneumoniae*, including the enhanced expression of resistance–nodulation–cell division (RND)-type efflux pumps such as AcrAB-TolC and OqxAB, mutations in the ribosomal S10 protein (encoded by *rpsJ* and *lon* genes; Ruzin et al., 2005; Villa et al., 2014; He et al., 2015; Fang et al., 2016), acquisition of plasmid-mediated *tmexCD1-toprJ1* efflux pump (Lv et al., 2020), mutation of *tet*(A) gene (Du et al., 2018).

In recently, four plasmid-borne *tet*(X) variants mediating tigecycline resistance [*tet*(X3), *tet*(X4), *tet*(X5), and *tet*(X6)] have been detected in various species of bacteria obtained from animals, animal-derived foods, humans and environment samples in China (He et al., 2019; Chen et al., 2020; Dong et al., 2022). Among these *tet*(X) variants, *tet*(X4) gene is widely identified in *E. coli* isolates from food animals (Li Y. et al., 2021; Yu et al., 2021; Wang et al., 2022). To date, only a few studies have reported *tet*(X4)-positive *K. pneumoniae* isolated from pork and clinical sources (Li et al., 2022; Liu et al., 2022). Overall, the epidemiology and characteristics of *tet*(X)s-positive *K. pneumoniae* isolates are not fully understood. Here, we investigated the prevalence of *tet*(X) genes in *K. pneumoniae* isolates and further described the *tet*(X4)-harboring plasmids in *K. pneumoniae* isolates.

## Materials and methods

### Sample collection and identification of *tet*(X)s-positive *Klebsiella pneumoniae* strains

To determine the prevalence of *tet*(X)s-positive *K. pneumoniae* strains in animal-associated and clinical samples, 921 samples from pigs and humans in China were collected (Supplementary Table S1). Specifically, a total of 590 samples from a pig farm (56 swine nasal swabs; Guangdong province, China) and a swine slaughter house (419 swine nasal swabs, 67 swine anal swabs, and 48 skin swabs of workers; Guangdong province, China) were collected from October to November 2020. Besides, 184 human anal swab specimens including 171 hospitalized patients and 13 healthy individuals from hospital A collected in 2019 were also included. All samples were subjected to selection on brain heart infusion (BHI) plates containing tigecycline (2 mg/L). After incubation at 37°C for 16–18 h, the tigecycline-resistant colonies were selected. Then detection of the *tet*(X) genes was carried out by PCR using specific primers in Supplementary Table S2. Species identification was achieved by Matrix-assisted laser desorption/ionization time-of-flight mass spectrometry (MALDI-TOF MS). In addition, 147 clinical nonduplicate *K. pneumoniae* isolates were collected from bloodstream samples in four hospitals (named as B, C, D, and E) located in Guangdong province in China during the period 2008–2018, and all strains were tested for the presence of *tet*(X) genes.

### Antimicrobial susceptibility testing

The susceptibility to tetracycline (TET), chloramphenicol (CHL), ciprofloxacin (CIP), ceftazidime (CAZ), gentamicin (GEN), amikacin (AMK), cefotaxime (CTX), trimethoprim-sulfamethoxazole (SXT), imipenem (IMP), ampicillin (AMP), fosfomycin (FOS), and rifampin (RIF) was determined by the agar dilution method according to the Clinical and Laboratory Standards Institute (CLSI) guidelines (CLSI, 2019). MICs of MICs of tigecycline (TGC) and colistin (CT) were determined by the broth dilution method according to the guidelines of EUCAST (2021). The *E. coil* ATCC25922 served as quality control.

### Plasmid conjugation

Conjugation experiments were performed to test the transferability of the *tet*(X4)-harboring plasmids, using a sodium azide resistant *E. coli* J53 as the recipient. Briefly, a culture of *tet*(X4)-producing isolates and the recipient strain *E. coli* J53 were mixed (ratio of 1:9) in BHI broth and subjected to incubation for 6–8 h. The mixture was then spread on BHI agar plates containing 100 mg/L sodium azide and 1 mg/L tigecycline to select transconjugants that had acquired the *tet*(X4)-harboring plasmid. Colonies that grew on selective plates after incubation for 16–24 h at 37°C were further confirmed by PCR and Sanger sequencing.

### *Galleria mellonella* infection model

The *Galleria mellonella* infection model was used to test virulence and pathogenesis of *tet*(X4)-positive *K. pneumoniae* strains, which was carried out as described previously with slight modifications (Buckner et al., 2018). Overnight cultures of *K. pneumoniae* strains were washed with phosphate-buffered saline (PBS) and further adjusted with PBS to concentrations of 1 × 10^6^ CFU/ml. The larvae were injected with 10 uL of bacterial solution and negative control groups were inoculated with 10 uL of PBS, the hvKP4 *K. pneumoniae* strain was used as the hypervirulent control (Gu et al., 2018). Then the larvae were incubated at 37°C in the dark and the survival rates of the larvae were recorded. Kaplan–Meier survival curves were plotted using GraphPad Prism, and the log rank (Mantel-Cox) test was used to analyze the significant difference (*p* < 0.05) of the survival rates in *G. mellonella* infection model.

### Genome sequencing and bioinformatics analysis

Genomic DNA of *tet*(X)s-positive *K. pneumoniae* isolates was extracted using the Qiagen Blood & Tissue kit (Qiagen, Hilden, Germany). DNA libraries were constructed with 350-bp paired-end fragments and sequenced using an Illumina HiSeq 2000 platform. In addition, the PacBio Sequel System was performed to long-read sequencing the TKPN_8 strain. The sequencing reads were assembled into contigs using SPAdes version 3.10 (Bankevich et al., 2012). Genome sequences were annotated using Prokka (version 1.13.3; Seemann, 2014). Multilocus sequence typing (MLST) of isolates was conducted using MLST v2.11 based on assembled contigs (Raisanen et al., 2020). The core genes of bacterial genomes were extracted and aligned using Roary (Page et al., 2015). RAxML v8.2.10 was used to construct a maximum likelihood phylogeny of the strains (Stamatakis, 2006), then the phylogenetic tree was visualized by iTOL (Letunic and Bork, 2016). The SNP (sequence-based) and core-genome-based MLST (cgMLST) strategies on BacWGSTdb 2.0 were used for source tracking bacterial pathogens, and the phylogenetic tree was generated and visualized by Grapetree (Feng et al., 2021). Capsule (K) loci was identified using Kaptive (Wick et al., 2018). To obtain the complete sequence of *tet*(X)s-carrying plasmid, we combined the sequencing data from the genomic DNA and the plasmids, and predicted gaps were closed by PCR and Sanger sequencing using primers listed in Supplementary Table S3. Plasmid replicons, insertion sequences, antimicrobial resistance determinants and virulence factors were determined using online tools.^1^ Easyfig was used to visualize the genetic comparisons.

## Results

### Identification of *Klebsiella pneumoniae* isolates harboring *tet*(X4)

In this study, the prevalence of *tet*(X)s-positive *K. pneumoniae* was 0.22% (2/921). The two *tet*(X4)-carrying strains, designated as TKPN_3 and TKPN_8, were collected from different swine nasal swabs (one was isolated from the pig farm; the other was isolated from a swine slaughter house). No other *tet*(X) variants were detected in *K. pneumoniae* strains.

**Figure 1:**
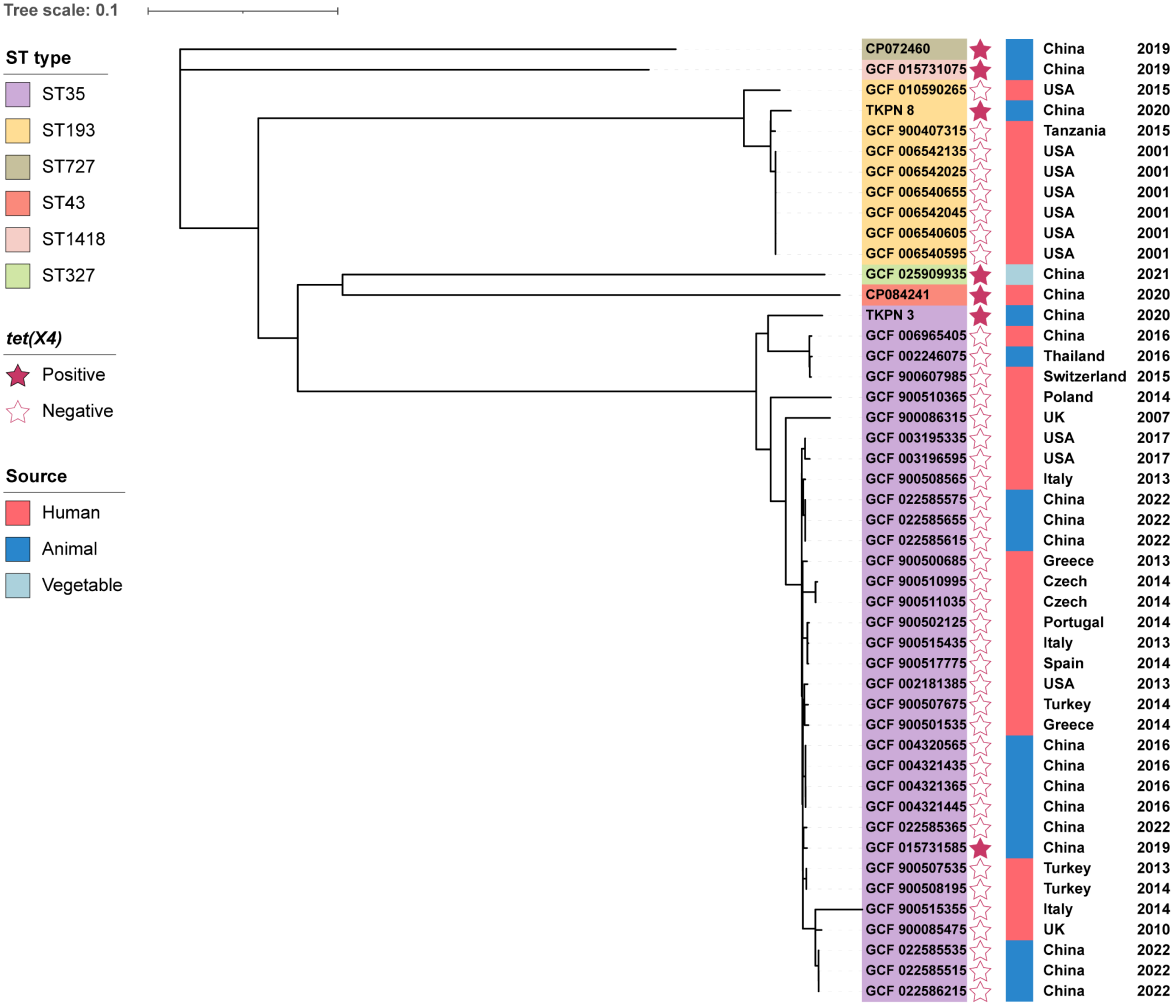
Phylogenetic analysis of 47 *K. pneumoniae* from different sources and their basic characterization. The phylogenetic tree was constructed by RAxML based on SNPs of core genomes.

The MLST analysis declared that *K. pneumoniae* TKPN_3 and TKPN_8 belong to ST35 and ST193, respectively (Table 1). To conduct the phylogenetic analysis of TKPN_3 and TKPN_8, the whole-genome sequencing data of 45 *K. pneumoniae* strains were download from NCBI database, including 40 *tet(X4)*-negative strains of ST35 and ST193, and five *tet(X4)*-positive strains belonging to ST727, ST43, ST1418, ST35, and ST327. A phylogenetic tree for 47 *K. pneumoniae* strains based on SNPs of core genomes was constructed. It showed that the *K. pneumoniae* strains were clustered into three distinct clusters, and *tet(X4)*-positive strains were dispersed on different branches, all of which were isolated from China between 2019 and 2021 (Figure 1). For source tracking bacterial pathogens, the genome sequences of TKPN_3 and TKPN_8 were submitted to BacWGSTdb and none close strains were found based on SNP (sequence-based) strategy. The core-genome-based MLST (cgMLST) analysis showed that 86 and 8 strains were, respectively, closed to TKPN_3 and TKPN_8, but had more than 340 different alleles (Supplementary Figure S1). In order to explore the evolution and transmission of the *tet*(X)s-positive *K. pneumoniae*, more sequencing data may be needed for phylogenetic analysis.

**Table 1:** Characteristics of the two *tet*(X4)-positive *Klebsiella pneumoniae* isolates.

			Transconjugant		Recipient
Characteristics	*K. pneumoniae* TKPN_3	*K. pneumoniae* TKPN_8	*E. Coli* J5*3-*pTKPN_3-186k-tetX4	*E. coli* J53-pTKPN_8-216k-tetX4	*E. coli* J53
Source	Pig farm	Swine slaughter house	/	/	This lab
Isolation site	Swine nasal swab	Swine nasal swab	/	/	/
Year	2020	2020	/	/	/
MLST	35	193	/	/	/
Capsular serotypes	KL71	KL63	/	/	/
Plasmid replicon type	ColRNAI, IncFIA(HI1), IncFIB(K), IncFIB(pKPHS1), IncFII, IncHI1A, IncHI1B(R27), IncQ1, IncQ2, IncX4	ColRNAI, IncFIA(HI1), IncFIB(K), IncFII, IncHI1A, IncHI1B(R27), IncR	IncHI1B(R27), IncHI1A, IncFIA(HI1)	IncHI1B(R27), IncHI1A, IncFIA(HI1)	/
MICs (mg/L)					
Tigecycline	256	32	32	32	0.25
Tetracycline	>256	256	128	128	≤1
Chloramphenicol	>128	>128	128	128	8
Ciprofloxacin	4	0.5	≤0.03	≤0.03	≤0.03
Colistin	0.5	0.5	0.25	0.25	≤0.125
Ceftazidime	32	≤1	≤1	≤1	≤1
Gentamicin	2	≤1	≤1	≤1	≤1
Cefotaxime	64	≤1	≤1	≤1	≤1
Trimethoprim-Sulfamethoxazole	>64	≤0.25	≤0.25	≤0.25	≤0.25
Fosfomycin	≤16	≤16	≤16	≤16	≤0.125
Amikacin	2	≤1	2	2	≤1
Ampicillin	>256	64	8	8	≤0.5
Rifampin	>128	16	16	16	≤0.5
Imipenem	1	≤0.25	0.5	0.5	0.5
Resistance genes					
Glycyrylcyclin	*tet*(X4)	*tet*(X4)	*tet*(X4)	*tet*(X4)	/
Aminoglycoside	*aac(6′)-Ib-cr*, *aadA16*, *aph(3″)-Ib*, *aph(6)-Id*, *ant(3″)-Ia*	*aph(3″)-Ib*, *aph(6)-Id*	/	/	/
Beta-lactam	*bla*_CTX-M-3_, *bla*_SHV-33_	*bla*_SHV-61_	/	/	/
Fosfomycin	*fosA6*	*fosA6*	/	/	/
Macrolide	*mph*(A)	/	/	/	/
Phenicol	*floR*	*floR*	/	/	/
Quinolone	*oqxB*, *oqxA*, *qnrS1*	*oqxB*, *oqxA*, *qnrS1*	/	/	/
Rifampicin	*ARR-3*	/	/	/	/
Sulphonamide	*sul2*	*sul2*	/	/	/
Tetracycline	*tet*(A)	*tet*(A)	/	/	/
Lincosamide	*lnu*(G)	*lnu*(G)	/	/	/
Trimethoprim	*dfrA27*	/	/	/	/

MLST, multilocus sequence typing.

### The antimicrobial resistance phenotype and genotype of *tet*(X4)-positive *Klebsiella pneumoniae*

*Klebsiella pneumoniae* TKPN_3 and TKPN_8 were resistant to tigecycline, tetracycline, rifampin, chloramphenicol and ampicillin, but were susceptible to gentamicin, imipenem, amikacin, fosfomycin and colistin. TKPN_3 and TKPN_8 showed resistance to tigecycline with MIC of 256 and 32 mg/L, respectively. In addition, TKPN_3 was also resistant to ciprofloxacin, ceftazidime, cefotaxime and trimethoprim-sulfamethoxazole, while TKPN_8 was susceptible to them (Table 1).

Except for the *tet*(X4) gene, WGS data revealed that TKPN_3 harbored other 18 antibiotic-resistant genes (ARGs), including three quinolone resistance genes (*oqxB*, *oqxA*, *qnrS1*), two β-lactams resistance genes (*bla*_CTX-M-3_, *bla*_SHV-33_), one sulphonamide resistance gene (*sul2*), one trimethoprim resistance gene (*dfrA27*), one rifampicin resistance gene (*arr-3*), one fosfomycin resistance gene (*fosA6*), one macrolide resistance gene [*mph*(A)], one tetracycline resistance gene [*tet*(A)], five aminoglycoside resistance genes [*aac(6′)-Ib-cr*, *aadA16*, *aph(3″)-Ib*, *aph(6)-Id*, *ant(3″)-Ia*], one chloramphenicol resistance gene (*floR*) and one lincomycin resistance gene [*lnu*(G)]. In the isolate TKPN_8, multiple resistance genes were also identified, including *tet*(X4) along with other 11 ARGs including three quinolone resistance gene (*oqxB*, *oqxA*, *qnrS1*), one β-lactam resistance gene (*bla*_SHV-61_), one sulphonamide resistance gene (*sul2*), one fosfomycin resistance gene (*fosA6*), one tetracycline resistance gene [*tet*(A)], two aminoglycoside resistance genes [*aph(3″)-Ib*, *aph(6)-Id*], chloramphenicol resistance gene (*floR*) and one lincomycin resistance gene [*lnu*(G); Table 1]. In general, these two *tet*(X4)-positive *K. pneumoniae* strains have similar antimicrobial resistance phenotype and genotype. Moreover, TKPN_3 showed higher level of tigecycline resistance compared with TKPN_8, and correspondingly, mutations involving regulators of multidrug efflux pump were also found in strain TKPN_3. Amino acid mutations were identified in RamR at T141I and in Lon at A299T.

### The virulence phenotype and genotype in *Klebsiella pneumoniae* TKPN_3 and TKPN_8

In addition to resistance genes, a total of 78 different virulence factors were identified among these two *tet*(X4) positive isolates. These genes include various siderophores such as enterobactin (encoded by *entABCEF*, *fepABCDG*, *fes*, and *ybdA* genes), aerobactin (encoded by *iucABCD* and *iutA* genes) and salmochelin (encoded by *iroE* gene). Moreover, the gene clusters *tssABCD* and *tssFGHIJKLM* encoding type VI secretion system, which was proved to be beneficial to bacterial competition, cell invasion, type-1 fimbriae expression and *in vivo* colonization in *K. pneumoniae* (Hsieh et al., 2019), were detected in TKPN_3 and TKPN_8. In addition, the capsular serotypes of the TKPN_3 and TKPN_8 strains were identified as KL71 and KL63, respectively. Then the virulence of two strains was demonstrated by *Galleria mellonella* infection model. After 12 h post-infection, the survival rate of *G. mellonella* larvaes injected with TKPN_3 and TKPN_8 was 50%. Overall, no difference in survival was observed for *G. mellonella* infected by TKPN_3 and TKPN_8 compared with HvKP4 (TKPN_3 vs. HvKP4, *p* = 0.606; TKPN_8 vs. HvKP4, *p* = 0.326; Supplementary Figure S2).

### The transferability of *tet*(X4)-harboring plasmid from *Klebsiella pneumoniae*

To further determine the transferability of *tet*(X4) gene, conjugation assays were conducted. It showed that the *tet*(X4)-carrying plasmids form TKPN_3 and TKPN_8 could be successfully transferred from *K. pneumoniae* to *E. coli* J53. The two *tet*(X4)-harboring plasmids were denoted as pTKPN_3-186k-tetX4 and pTKPN_8-216k-tetX4. The conjugation frequencies of pTKPN_3-186k-tetX4 and pTKPN_8-216k-tetX4 were (1.0 ± 0.1) × 10^−4^ and (5.4 ± 0.7) × 10^−5^ cells per recipient, respectively. Compared with the recipient strain *E. coli* J53, the MICs of the transconjugants J53/pTKPN_3-186k-tetX4 and J53/pTKPN_8-216k-tetX4 for tigecycline and tetracycline increased by 128-fold, respectively.

### Genetic characteristics of *tet*(X4) in pTKPN_3-186k-tetX4 and pTKPN_8-216k-tetX4

**Figure 2:**
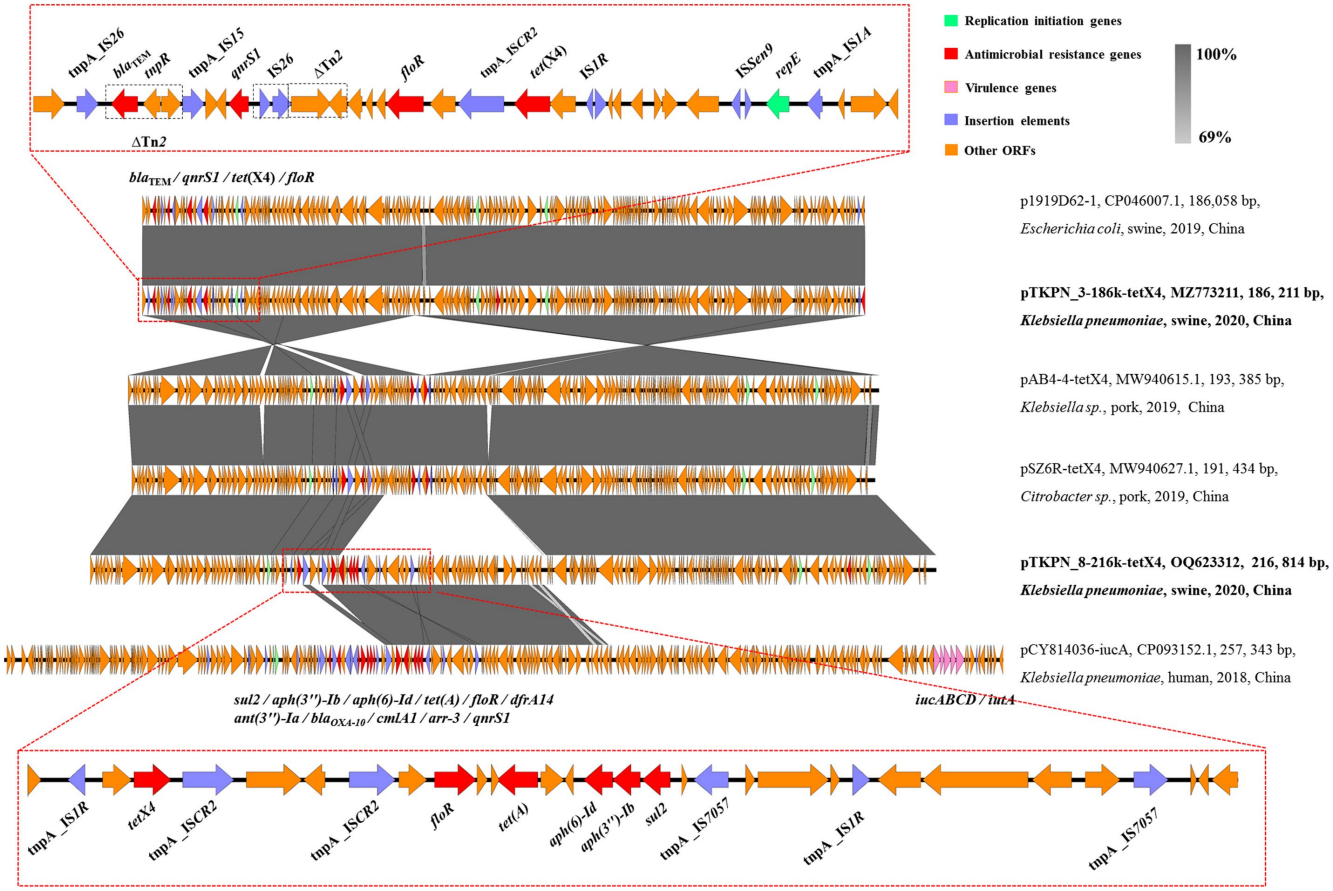
Structure analysis of plasmids pTKPN_3-186k-tetX4 and pTKPN_8-216k-tetX4. Major structural features of pTKPN_3-186k-tetX4 and pTKPN_8-216k-tetX4 were compared with those of plasmids p1919D62-1 (GenBank accession no. CP046007.1), pAB4-4-tetX4 (GenBank accession no. MW940615.1), pSZ6R-tetX4 (GenBank accession no. MW940627.1), and pCY814036-iucA (GenBank accession no. CP093152.1). Gray shading indicates shared regions with a high degree of homology (>69%). Red and pink represent the antimicrobial resistance genes and virulence genes, respectively, and purple is the insertion elements.

The genetic features of *tet*(X4)-carrying plasmid pTKPN_3-186k-tetX4 of TKPN_3 was analyzed in depth. To obtain the complete sequence of this plasmid, the sequencing data of the genomic DNA and the plasmids DNA were combined, and predicted gaps were closed by PCR and Sanger sequencing. pTKPN_3-186k-tetX4 was a 186,211 bp plasmid and encoded 218 predicted open reading frames (ORFs). The backbone of the *tet*(X4)-carrying plasmid pTKPN_3-186k-tetX4 showed a typical mosaic structure with multiple replicon types including IncHI1B(R27), IncHI1A and IncFIA (HI1). The genes involved in conjugation were identified including *traC*, *traD*, *traG*, *traI*, *traJ*, *traU*, and *mobI*. The *tet*(X4) gene and another five antimicrobial resistance genes *bla*_TEM-1B_, *ant(3″)-Ia*, *qnrS1*, *floR*, and *lnu*(G) were found in pTKPN_3-186k-tetX4. The mobile element IS*1R* (IS*1* family, 768 bp) was located upstream of *tet*(X4) and delta IS*CR2* (IS*91* family, 977 bp) was found in downstream of *tet*(X4), which may contribute to the transmission of the *tet*(X4) gene (Figure 2). Additionally, two complete insertion sequence IS*26* and two truncated transposons Tn*2* were identified downstream of *tet*(X4) in pTKPN_3-186k-tetX4. The pTKPN_3-186k-tetX4 shared a similar plasmid backbone (99% coverage and 100% identity) against p1919D62-1 (GenBank accession number: CP046007.1), which isolated from a swine *E.coli* strain 1919D62 in China. The other two plasmids with high similarity with our plasmid were pSZ6R-tetX4 (GenBank accession number: MW940627.1) and pAB4-4-tetX4 (GenBank accession number: MW940615.1). The two plasmids pSZ6R-tetX4 and pAB4-4-tetX4 were isolated from *Citrobacter* sp. and *Klebsiella sp*. strains, respectively, (Figure 2).

The complete sequence of another *tet*(X4)-positive plasmid pTKPN_8-216k-tetX4 was obtained by long-read sequencing. pTKPN_8-216k-tetX4 was 216,814 bp in size and also belongs to an IncHI1B(R27)/IncHI1A/IncFIA(HI1) type hybrid plasmid. pTKPN_8-216k-tetX4 exhibited a high similarity (74% coverage, 100% identity) with pTKPN_3-186k-tetX4, in particular, it had an additional ~30 kp fragment. This fragment was similar to pCY814036-iucA (GenBank accession number: CP093152.1; Figure 2). pCY814036-iucA was a multi-drug resistant and hypervirulent hybrid plasmid and isolated from a *K. pneumoniae* strain in China. pTKPN_8-216k-tetX4 also carried the conjugation-related genes *traC*, *traD*, *traG*, *traI*, *traJ*, *traU*, and *mobI*. In addition to *tet(X4)* gene, *aph(3″)-Ib*, *aph(6)-Id*, *floR*, *sul2* and *tet(A)* were identified in pTKPN_8-216k-tetX4. It was worth noting that similar to pTKPN_3-186k-tetX4, *tet(X4)* gene was flanked by mobile elements IS*1R* and IS*CR2* in pTKPN_8-216k-tetX4 (Figure 2).

## Discussion

To date, the plasmid-borne *tet*(X) genes have been identified in >15 different Gram-negative species, with *tet*(X4)-positive *E. coli* isolated from food-producing animals being the most common (He et al., 2019; Sun et al., 2019). The *tet*(X4)-carrying *K. pneumoniae* strains has so far been rarely reported. A recently investigation showed 58 *tet*(X4)-positive strains were isolated from 139 fresh pork samples and the most strains were identified as *E. coli* (55/58), only two were *K. pneumoniae* (Li R. et al., 2021). In this study, two *tet*(X4)-harbouring tigecycline resistant *K. pneumoniae* were detected from swine nasal swabs in China. According to previous study, the prevalence of *tet*(X) genes in *K. pneumoniae* was significantly lower than in *Escherichia coli* (3.84%, 95/2475) and *Acinetobacter* spp. (5.02%, 193/3846; Chen et al., 2020; Sun et al., 2020). Although the *tet*(X4) gene was present in *K. pneumoniae* at a low prevalence, the TKPN_3 and TKPN_8 strains also contained many important virulence factors (Hsieh et al., 2019; Wang et al., 2020), such as enterobactin and aerobactin, and showed high virulence phenotype. Given the harmfulness of *K. pneumoniae* in clinical infections, the emergence of a *tet*(X4)-positive strain should be alarmed.

Until now, *tet*(X4)-bearing plasmids ranged from 9 to 315 kb and were categorized as ColE2-like, IncQ, IncX1, IncX4, IncA/C2, IncFII, IncFIB, IncI1, and hybrid plasmids with different replicons (Li R. et al., 2020), which suggested that *tet*(X4) gene could be captured by a range of mobile genetic elements circulating among bacterial strains. Moreover, the mobile elements, particularly IS*CR2* and IS*1R*, could assist the integration and spread of *tet*(X4) gene between different plasmids. The host range of the *tet*(X4) gene is likely to be further expanded. According to *tet*(X4)-bearing plasmid type distribution, IncFIB(K)/IncFIA(HI1)/IncX1 hybrid plasmids were the most widespread in the sequenced plasmids (Li R. et al., 2020). In this study, both pTKPN_3-186k-tetX4 and pTKPN_8-216k-tetX4 belonged to IncHI1B(R27)/IncHI1A/IncFIA(HI1) hybrid plasmid type, which have been reported in several different species such as *E. coli*, *Salmonella enterica* and *Citrobacter* sp. Correspondingly, pTKPN_3-186k-tetX4 and pTKPN_8-216k-tetX4 was confirmed to be able to transfer from *K. pneumoniae* to *E. coli*. In contrast, IncFII-type plasmid carrying *tet*(X4) in *K. pneumoniae* was found to be non-self-transferable and could be only co-transferred with the help of other conjugative plasmids (Zhai et al., 2022). It is possible that pTKPN_3-186k-tetX4-like plasmids will become more widespread in the near future.

Worrisomely, the *tet*(X)-mediated tigecycline resistance has been detected in carbapenem- and colistin-resistant *Acinetobacter* spp. and *Escherichia* spp. strains (Chen et al., 2020; Li Y. et al., 2020). Future efforts are needed to prevent and monitor the emergence of *tet*(X)-mediated tigecycline- and carbapenem- resistant *K. pneumoniae* from all related sectors. Our data contributes to understanding of the genetic characteristic of *tet*(X4) and their transferabilities in *K. pneumoniae*.

## Data availability statement

The names of the repository/repositories and accession number(s) can be found at: https://www.ncbi.nlm.nih.gov/genbank/, MZ773211 and OQ623312.

## Author contributions

L-NQ, BY, and MD: conceptualization. YY and RH: data curation. YY, RH, YW, and MQ: investigation. YY, RH, YW, MQ, JC, YF, RZ, LX, and XG: methodology. G-BT, L-NQ, BY, and MD: resources. YY and RH: visualization. YY, RH, YW, and MQ: writing original draft. L-NQ, BY, MD, and G-BT: writing, review and editing. All authors contributed to the article and approved the submitted version.

## Funding

This work was supported by the National Natural Science Foundation of China (grant number 82061128001 and 81830103 to G-BT), Guangdong Natural Science Foundation (grant number 2017A030306012 to G-BT), National Key Research and Development Program (grant number 2017ZX10302301 to G-BT), Project of High-level Health Teams of Zhu hai in 2018 (The Innovation Team for Antimicrobial Resistance and Clinical Infection to G-BT), and the Project 111 (grant number B12003 to G-BT).

## Conflict of interest

The authors declare that the research was conducted in the absence of any commercial or financial relationships that could be construed as a potential conflict of interest.

## Publisher’s note

All claims expressed in this article are solely those of the authors and do not necessarily represent those of their affiliated organizations, or those of the publisher, the editors and the reviewers. Any product that may be evaluated in this article, or claim that may be made by its manufacturer, is not guaranteed or endorsed by the publisher.

## Footnotes

^1^ https://cge.cbs.dtu.dk/services/

## References

[ref1] Bankevich A. Nurk S. Antipov D. Gurevich A. A. Dvorkin M. Kulikov A. S.. (2012). SPAdes: a new genome assembly algorithm and its applications to single-cell sequencing. J. Comput. Biol. 19, 455–477. doi: 10.1089/cmb.2012.0021, PMID: 22506599 PMC3342519

[ref2] Buckner M. M. C. Saw H. T. H. Osagie R. N. Mcnally A. Ricci V. Wand M. E.. (2018). Clinically relevant plasmid-host interactions indicate that transcriptional and not genomic modifications ameliorate fitness costs of *Klebsiella pneumoniae* carbapenemase-carrying plasmids. MBio 9, e02303–e02317. doi: 10.1128/mBio.02303-17 PMC5915730 29691332

[ref3] Chen C. Cui C. Y. Yu J. J. He Q. Wu X. T. He Y. Z.. (2020). Genetic diversity and characteristics of high-level tigecycline resistance *Tet(X)* in Acinetobacter species. Genome Med. 12:111. doi: 10.1186/s13073-020-00807-5, PMID: 33287863 PMC7722449

[ref4] CLSI (2019). Performance standards for antimicrobial susceptibility testing, 29th. Annapolis Junction, MD: Clinical and Laboratory Standards Institute.

[ref5] Dong N. Zeng Y. Cai C. Sun C. Lu J. Liu C.. (2022). Prevalence, transmission, and molecular epidemiology of *tet(X)*-positive bacteria among humans, animals, and environmental niches in China: an epidemiological, and genomic-based study. Sci. Total Environ. 818:151767. doi: 10.1016/j.scitotenv.2021.151767, PMID: 34801490

[ref6] Du X. He F. Shi Q. Zhao F. Xu J. Fu Y.. (2018). The rapid emergence of tigecycline resistance in *bla*_KPC-2_ harboring *Klebsiella pneumoniae*, as mediated in vivo by mutation in *tetA* during tigecycline treatment. Front. Microbiol. 9:648. doi: 10.3389/fmicb.2018.00648, PMID: 29675006 PMC5895649

[ref7] EUCAST (2021). European committee on antimicrobial susceptibility testing breakpoint tables for interpretation of MICs and zone diameters European committee on antimicrobial susceptibility testing breakpoint tables for interpretation of MICs and zone diameters, version 11.0. Sweden: EUCAST.

[ref8] Fang L. Chen Q. Shi K. Li X. Shi Q. He F.. (2016). Step-wise increase in tigecycline resistance in *Klebsiella pneumoniae* associated with mutations in *ramR*, lon and rpsJ. PLoS One 11:e0165019. doi: 10.1371/journal.pone.0165019, PMID: 27764207 PMC5072711

[ref9] Feng Y. Zou S. Chen H. Yu Y. Ruan Z. (2021). BacWGSTdb 2.0: a one-stop repository for bacterial whole-genome sequence typing and source tracking. Nucleic Acids Res. 49, D644–D650. doi: 10.1093/nar/gkaa821, PMID: 33010178 PMC7778894

[ref10] Gu D. Dong N. Zheng Z. Lin D. Huang M. Wang L.. (2018). A fatal outbreak of ST11 carbapenem-resistant hypervirulent *Klebsiella pneumoniae* in a Chinese hospital: a molecular epidemiological study. Lancet Infect. Dis. 18, 37–46. doi: 10.1016/s1473-3099(17)30489-9, PMID: 28864030

[ref11] Hansen D. S. Gottschau A. Kolmos H. J. (1998). Epidemiology of *Klebsiella bacteraemia*: a case control study using Escherichia coli bacteraemia as control. J. Hosp. Infect. 38, 119–132. doi: 10.1016/s0195-6701(98)90065-2 9522290

[ref12] He F. Fu Y. Chen Q. Ruan Z. Hua X. Zhou H.. (2015). Tigecycline susceptibility and the role of efflux pumps in tigecycline resistance in KPC-producing *Klebsiella pneumoniae*. PLoS One 10:e0119064. doi: 10.1371/journal.pone.0119064, PMID: 25734903 PMC4348519

[ref13] He T. Wang R. Liu D. Walsh T. R. Zhang R. Lv Y.. (2019). Emergence of plasmid-mediated high-level tigecycline resistance genes in animals and humans. Nat. Microbiol. 4, 1450–1456. doi: 10.1038/s41564-019-0445-2 31133751

[ref14] Hsieh P. F. Lu Y. R. Lin T. L. Lai L. Y. Wang J. T. (2019). *Klebsiella pneumoniae* type VI secretion system contributes to bacterial competition, cell invasion, type-1 fimbriae expression, and in vivo colonization. J. Infect. Dis. 219, 637–647. doi: 10.1093/infdis/jiy534, PMID: 30202982 PMC6350951

[ref15] Letunic I. Bork P. (2016). Interactive tree of life (iTOL) v3: an online tool for the display and annotation of phylogenetic and other trees. Nucleic Acids Res. 44, W242–W245. doi: 10.1093/nar/gkw290, PMID: 27095192 PMC4987883

[ref16] Li Y. Li Y. Bu K. Wang M. Wang Z. Li R. (2022). Antimicrobial resistance and genomic epidemiology of tet(X4)-bearing bacteria of pork origin in Jiangsu, China. Genes 14:36. doi: 10.3390/genes14010036, PMID: 36672777 PMC9858217

[ref17] Li R. Li Y. Peng K. Yin Y. Liu Y. He T.. (2021). Comprehensive genomic investigation of tigecycline resistance gene *tet(X4)*-bearing strains expanding among different settings. Microbiol. Spectr. 9:e0163321. doi: 10.1128/spectrum.01633-21, PMID: 34937176 PMC8694195

[ref18] Li R. Lu X. Peng K. Liu Z. Li Y. Liu Y.. (2020). Deciphering the structural diversity and classification of the mobile tigecycline resistance gene *tet(X)*-bearing plasmidome among bacteria. mSystems 5, e00134–e00120. doi: 10.1128/mSystems.00134-20, PMID: 32345737 PMC7190383

[ref19] Li Y. Wang Q. Peng K. Liu Y. Li R. Wang Z. (2020). Emergence of carbapenem- and tigecycline-resistant proteus cibarius of animal origin. Front. Microbiol. 11:1940. doi: 10.3389/fmicb.2020.01940, PMID: 32922378 PMC7457074

[ref20] Li Y. Wang Q. Peng K. Liu Y. Xiao X. Mohsin M.. (2021). Distribution and genomic characterization of tigecycline-resistant *tet(X4)*-positive *Escherichia coli* of swine farm origin. Microb. Genom. 7:000667. doi: 10.1099/mgen.0.000667, PMID: 34693904 PMC8627205

[ref21] Liu C. Dong N. Zeng Y. Lu J. Chen J. Wang Y.. (2022). Co-transfer of last-line antibiotic resistance and virulence operons by an IncFIBk-FII-X3-ColKP3 hybrid plasmid in *Klebsiella pneumoniae*. J. Antimicrob. Chemother. 77, 1856–1861. doi: 10.1093/jac/dkac121 35445265

[ref22] Low Y. M. Chong C. W. Yap I. K. S. Chai L. C. Clarke S. C. Ponnampalavanar S.. (2018). Elucidating the survival and response of carbapenem resistant *Klebsiella pneumoniae* after exposure to imipenem at sub-lethal concentrations. Pathog. Glob. Health 112, 378–386. doi: 10.1080/20477724.2018.1538281, PMID: 30380366 PMC6300736

[ref23] Lv L. Wan M. Wang C. Gao X. Yang Q. Partridge S. R.. (2020). Emergence of a plasmid-encoded resistance-nodulation-division efflux pump conferring resistance to multiple drugs, including tigecycline, in *Klebsiella pneumoniae*. MBio 11, e02930–e02919. doi: 10.1128/mBio.02930-19 32127452 PMC7064769

[ref24] Page A. J. Cummins C. A. Hunt M. Wong V. K. Reuter S. Holden M. T.. (2015). Roary: rapid large-scale prokaryote pan genome analysis. Bioinformatics 31, 3691–3693. doi: 10.1093/bioinformatics/btv421, PMID: 26198102 PMC4817141

[ref25] Raisanen K. Lyytikainen O. Kauranen J. Tarkka E. Forsblom-Helander B. Gronroos J. O.. (2020). Molecular epidemiology of carbapenemase-producing *Enterobacterales* in Finland, 2012-2018. Eur. J. Clin. Microbiol. Infect. Dis. 39, 1651–1656. doi: 10.1007/s10096-020-03885-w, PMID: 32307627 PMC7427707

[ref26] Ruzin A. Visalli M. A. Keeney D. Bradford P. A. (2005). Influence of transcriptional activator RamA on expression of multidrug efflux pump AcrAB and tigecycline susceptibility in *Klebsiella pneumoniae*. Antimicrob. Agents Chemother. 49, 1017–1022. doi: 10.1128/AAC.49.3.1017-1022.2005, PMID: 15728897 PMC549240

[ref27] Seemann T. (2014). Prokka: rapid prokaryotic genome annotation. Bioinformatics 30, 2068–2069. doi: 10.1093/bioinformatics/btu153, PMID: 24642063

[ref28] Seifert H. Blondeau J. Dowzicky M. J. (2018). In vitro activity of tigecycline and comparators (2014-2016) among key WHO 'priority pathogens' and longitudinal assessment (2004-2016) of antimicrobial resistance: a report from the T.E.S.T. study. Int. J. Antimicrob. Agents 52, 474–484. doi: 10.1016/j.ijantimicag.2018.07.003, PMID: 30012439

[ref29] Siu L. K. Yeh K. M. Lin J. C. Fung C. P. Chang F. Y. (2012). *Klebsiella pneumoniae* liver abscess: a new invasive syndrome. Lancet Infect. Dis. 12, 881–887. doi: 10.1016/S1473-3099(12)70205-0, PMID: 23099082

[ref30] Stamatakis A. (2006). RAxML-VI-HPC: maximum likelihood-based phylogenetic analyses with thousands of taxa and mixed models. Bioinformatics 22, 2688–2690. doi: 10.1093/bioinformatics/btl446, PMID: 16928733

[ref31] Sun J. Chen C. Cui C. Y. Zhang Y. Liu X. Cui Z. H.. (2019). Plasmid-encoded tet(X) genes that confer high-level tigecycline resistance in *Escherichia coli*. Nat. Microbiol. 4, 1457–1464. doi: 10.1038/s41564-019-0496-4, PMID: 31235960 PMC6707864

[ref32] Sun C. Cui M. Zhang S. Liu D. Fu B. Li Z.. (2020). Genomic epidemiology of animal-derived tigecycline-resistant *Escherichia coli* across China reveals recent endemic plasmid-encoded *tet(X4)* gene. Commun. Biol. 3:412. doi: 10.1038/s42003-020-01148-0, PMID: 32737421 PMC7395754

[ref33] Villa L. Feudi C. Fortini D. Garcia-Fernandez A. Carattoli A. (2014). Genomics of KPC-producing *Klebsiella pneumoniae* sequence type 512 clone highlights the role of RamR and ribosomal S10 protein mutations in conferring tigecycline resistance. Antimicrob. Agents Chemother. 58, 1707–1712. doi: 10.1128/AAC.01803-13, PMID: 24379204 PMC3957836

[ref34] Wang J. Lu M. J. Wang Z. Y. Jiang Y. Wu H. Pan Z. M.. (2022). Tigecycline-resistant *Escherichia coli* ST761 carrying *tet(X4)* in a pig farm, China. Front. Microbiol. 13:967313. doi: 10.3389/fmicb.2022.967313, PMID: 36016796 PMC9396132

[ref35] Wang G. Zhao G. Chao X. Xie L. Wang H. (2020). The characteristic of virulence, biofilm and antibiotic resistance of *Klebsiella pneumoniae*. Int. J. Environ. Res. Public Health 17:6278. doi: 10.3390/ijerph17176278, PMID: 32872324 PMC7503635

[ref36] Wick R. R. Heinz E. Holt K. E. Wyres K. L. (2018). Kaptive web: user-friendly capsule and lipopolysaccharide serotype prediction for *Klebsiella* genomes. J. Clin. Microbiol. 56, e00197–e00118. doi: 10.1128/JCM.00197-18, PMID: 29618504 PMC5971559

[ref37] Wyres K. L. Lam M. M. C. Holt K. E. (2020). Population genomics of *Klebsiella pneumonia*e. Nat. Rev. Microbiol. 18, 344–359. doi: 10.1038/s41579-019-0315-1 32055025

[ref38] Yu Y. Cui C. Y. Kuang X. Chen C. Wang M. G. Liao X. P.. (2021). Prevalence of *tet(X4)* in *Escherichia coli* from duck farms in Southeast China. Front. Microbiol. 12:716393. doi: 10.3389/fmicb.2021.716393, PMID: 34497596 PMC8419466

[ref39] Zhai W. Tian Y. Lu M. Zhang M. Song H. Fu Y.. (2022). Presence of mobile tigecycline resistance gene tet(X4) in clinical *Klebsiella pneumoniae*. Microbiol. Spectr. 10:e0108121. doi: 10.1128/spectrum.01081-21, PMID: 35138117 PMC8826827

