# Supplementary material for: Characterization of two multidrug-resistant Klebsiella pneumoniae harboring tigecycline-resistant gene tet(X4) in China
Source: Front Microbiol. 2023 Apr 26;14:1130708. doi: 10.3389/fmicb.2023.1130708 (PMC10171367; doi:10.3389/fmicb.2023.1130708)
Supplement: Supplementary file 1 [file Data_Sheet_1.docx]

**Table S1 Samples collection and bacteria.**

| **Source** | **Samples Types** | | | |
| --- | --- | --- | --- | --- |
|  | **swine nasal swabs** | **anal swabs** | **skin swabs of workers** | **Blood** |
| Pig farm | 56 | NA | NA | NA |
| Swine slaughter house | 419 | 67 | 48 | NA |
| Human specimens from hospital A | NA | 184 | NA | NA |
| K. pneumoniae strains from hospital B, C, D and E | NA | NA | NA | 147 |

**Table S2 Primers used to amplify *tet*(X)s genes.**

| **Primers** | **Sequence (5' to 3')** | **References** |
| --- | --- | --- |
| tet(X3)-F | CAACGACCGAGAGGCAAGAA | In this study |
| tet(X3)-R | TTGCGTGTCGGTAACAAAGC |  |
| tet(X4)-F | AGGAACAGGACACGAATTGC | ([Ding et al., 2020](#_ENREF_1)) |
| tet(X4)-R | TTACTGGCGGAGCCGTCTA |  |
| tet(X5)-F | TGGACCCGTTGGACTGACTA | ([Ji et al., 2020](#_ENREF_3)) |
| tet(X5)-R | AGGCATCCATCAACCCACTG |  |
| tet(X6)-F | GGCGAGCTCATGACTTTACTAAAACATAA | ([He et al., 2020](#_ENREF_2)) |
| tet(X6)-R | GCGTCTAGATTATAGATTCATTAGTTTTTGG |  |

**References**

Ding, Y., Saw, W.Y., Tan, L.W.L., Moong, D.K.N., Nagarajan, N., Teo, Y.Y., et al. (2020). Emergence of tigecycline- and eravacycline-resistant Tet(X4)-producing Enterobacteriaceae in the gut microbiota of healthy Singaporeans. *J Antimicrob Chemother* 75**,** 3480-3484.doi:10.1093/jac/dkaa372

He, D., Wang, L., Zhao, S., Liu, L., Liu, J., Hu, G., et al. (2020). A novel tigecycline resistance gene, tet(X6), on an SXT/R391 integrative and conjugative element in a Proteus genomospecies 6 isolate of retail meat origin. *J Antimicrob Chemother* 75**,** 1159-1164.doi:10.1093/jac/dkaa012

Ji, K., Xu, Y., Sun, J., Huang, M., Jia, X., Jiang, C., et al. (2020). Harnessing efficient multiplex PCR methods to detect the expanding Tet(X) family of tigecycline resistance genes. *Virulence* 11**,** 49-56.doi:10.1080/21505594.2019.1706913

**Table S3 Primers used to amplify plasmid sequences.**

| **Plasmid** | **Primers** | **Sequence (5' to 3')** | **Product length (bp)** | **Position (bp)** |
| --- | --- | --- | --- | --- |
| pTKPN_3-186k-tetX4 | pTKPN3-10758-F | CACGCTGTCACGTTCATCAC | 552 | 10588-11141 |
|  | pTKPN3-10758-R | CTAGCAACGGCAGGCATTTC |  |  |
|  | pTKPN3-13618-F | GGCATCGACATAGCCCTCAT | 622 | 13568-14195 |
|  | pTKPN3-13618-R | TCAACCTAACCCGCTTCCAC |  |  |
|  | pTKPN3-14377-F | TTCGAAAATGACGTGCGTGG | 706 | 14252-14960 |
|  | pTKPN3-14377-R | TGGGTGTTGAGTTTCCCGTT |  |  |

**Figure S1** The phylogenetic trees of *K. pneumoniae* TKPN_3 (A) and TKPN_8 (B) based on core-genome-based MLST (cgMLST) analysis using BacWGSTdb 2.0 (threshold 1000). The tree was generated and visualized by Grapetree.

**Figure S2** Survival curves of *Galleria mellonella* larvae infected with TKPN_3, TKPN_8 and hvKP4 at final concentrations of 1×10^6^ colony-forming units (CFUs)/larva. Negative control groups included larvae that were inoculated with 10 μL of PBS. Experiments were performed three times on separate occasions.
