# Supplementary figures and images for: Characterization of two multidrug-resistant Klebsiella pneumoniae harboring tigecycline-resistant gene tet(X4) in China
Source: Front Microbiol. 2023 Apr 26;14:1130708. doi: 10.3389/fmicb.2023.1130708 (PMC10171367; doi:10.3389/fmicb.2023.1130708)

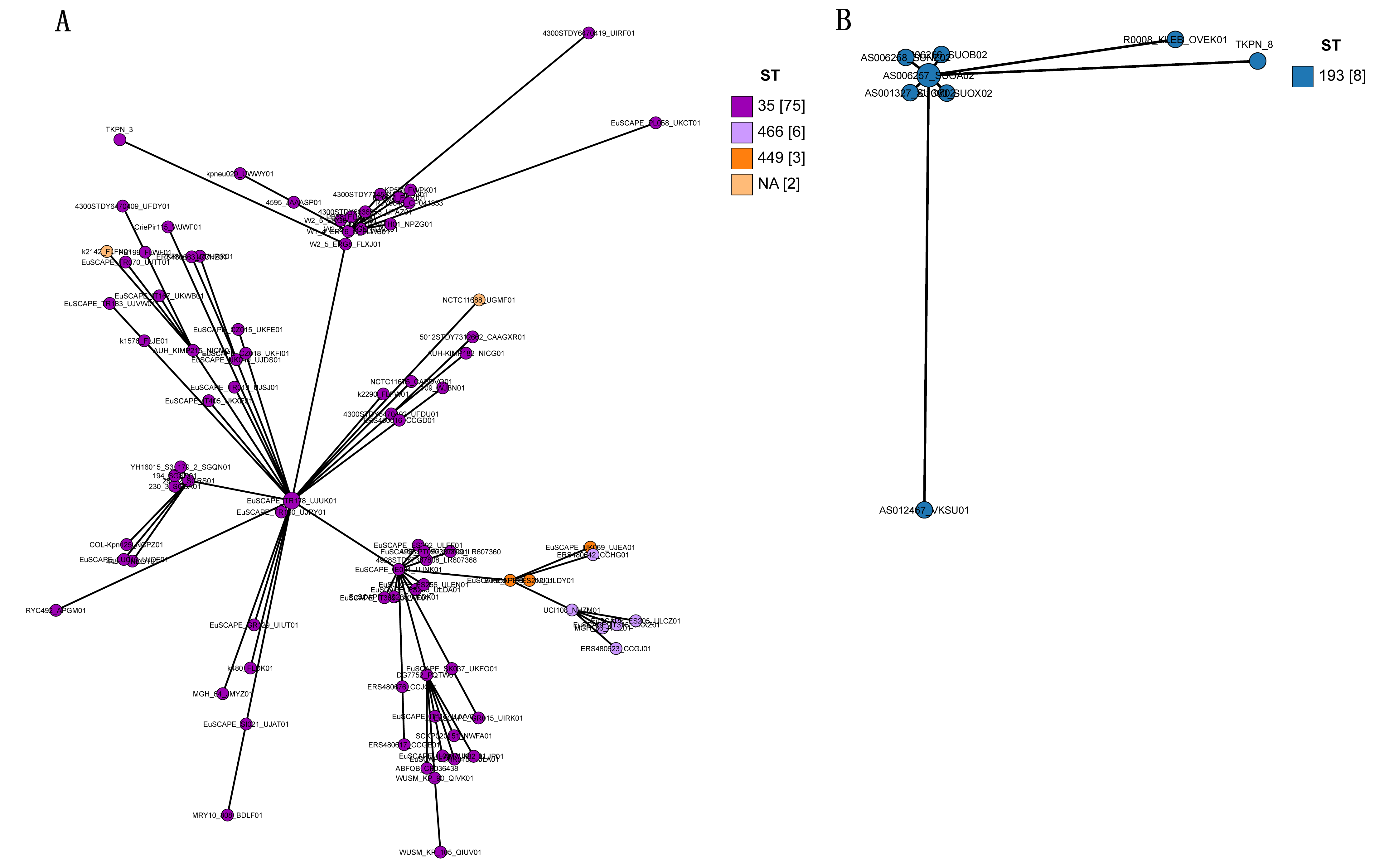

Supplement: Supplementary file 2 [file Image_1.TIF]

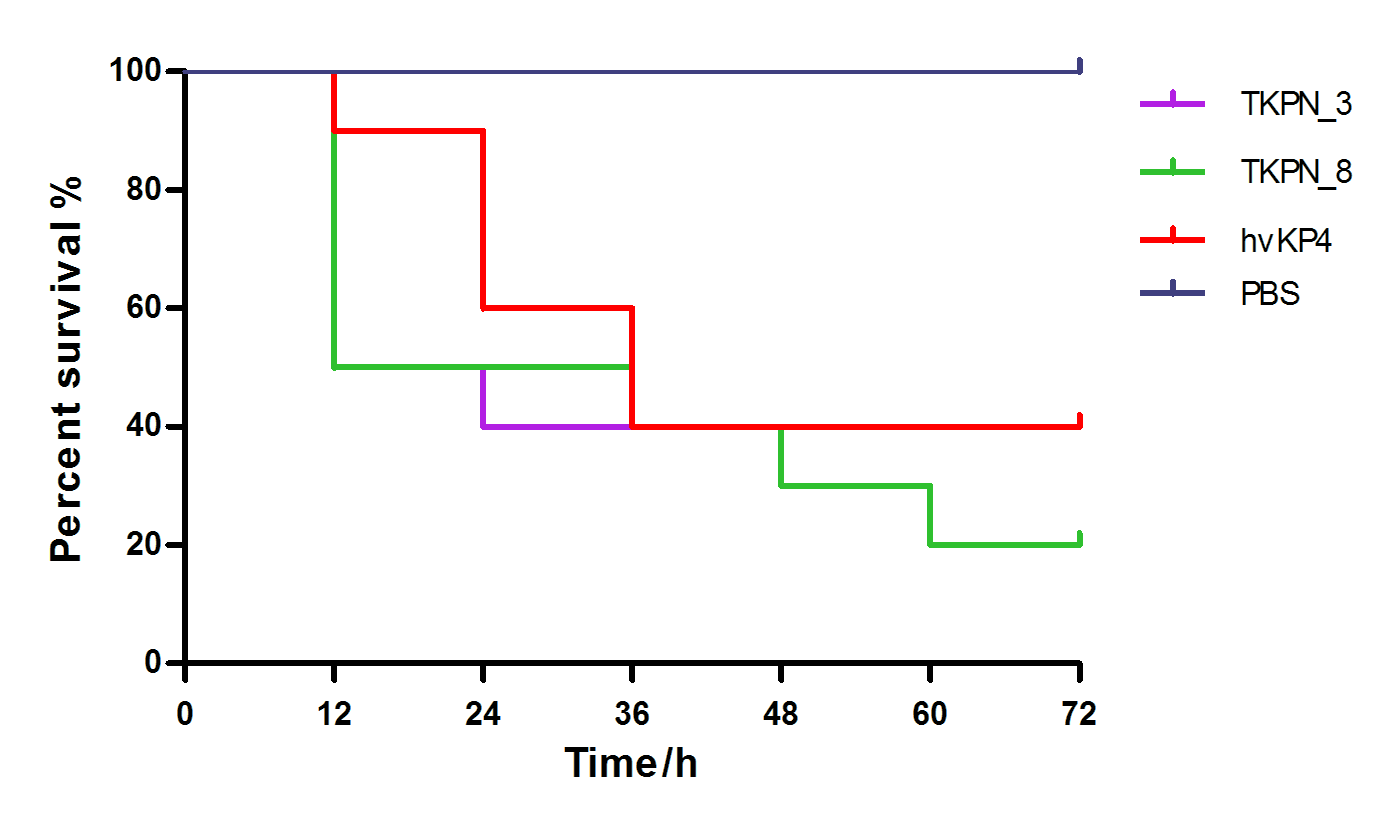

Supplement: Supplementary file 3 [file Image_2.TIF]
